# An AI-Assisted Workflow for Tea Product Discrimination and Moisture Prediction Using Portable NIR Spectroscopy

**DOI:** 10.3390/foods15213855

**Published:** 2026-10-28

**Authors:** Zhengfei Luo, Bo Zhao, Qing Meng, Dongling Qiao, Yanhong Liu, Guohua Zhao, Jia Chen

**Affiliations:** 1 Chinese-Hungarian Cooperative Research Centre for Food Science, College of Food Science, Southwest University, Chongqing 400715, China; luophy@126.com (Z.L.); qdttkl@163.com (D.Q.); 2 Yunnan Key Laboratory of Tea Germplasm Conservation and Utilization in the Lancang River Basin, College of Biotechnology and Engineering, West Yunnan University, Lincang 677000, China; liu41294777@126.com; 3 Key Laboratory of Condiment Supervision Technology, State Administration for Market Regulation, Chongqing Institute for Food and Drug Control, Chongqing 400731, China; zhaobocq@163.com; 4 Integrative Science Center of Germplasm Creation in Western China (Chongqing) Science City, College of Food Science, Southwest University, Chongqing 400715, China; 5 College of Life Sciences, Sichuan Normal University, Chengdu 610101, China; zhaogh@sicnu.edu.cn

**Keywords:** near-infrared spectroscopy, portable spectrometer, tea, product discrimination, moisture prediction, vibe coding, NIR-analysis skill

## Abstract

Portable near-infrared (NIR) spectrometers enable rapid tea analysis, but the heterogeneity of tea leaves can reduce spectral representativeness and modelling reliability. This study evaluated the influence of sampling configurations on tea product discrimination and moisture prediction using two portable NIR spectrometers and developed a domain-specific NIR-analysis skill to support the analytical workflow. Ten tea products were analysed in ground and unground states using the NIR-R210 and NeoSpectra Scanner. Sixteen preprocessing methods were evaluated with partial least squares and support vector machine models. Classification and regression models were selected using stratified four-fold and leave-one-product-out cross-validation, respectively. Within the investigated product and batch range, the NeoSpectra Scanner achieved 100% classification accuracy for both sample states in validation, whereas the NIR-R210 achieved 100% and 92.5%, respectively. Support vector regression achieved the best moisture-prediction validation performance for unground NeoSpectra Scanner samples (R^2^ = 0.95; RMSEP = 0.330%). External validation showed decreased performance, particularly for unground NIR-R210 samples (classification accuracy, 80.0%; R^2^ = 0.68; RMSEP = 0.797%). Grinding reduced spectral variability, particularly for the NIR-R210, while the NeoSpectra Scanner’s rotating sampling configuration may have contributed to more representative measurements of intact leaves. These findings highlight the importance of representative sampling for heterogeneous tea materials.

## 1. Introduction

Tea is one of the most widely consumed non-alcoholic beverages worldwide. Rapid tea quality assessment is of great importance in some special scenarios, such as quality control [1], storage management [2] and market surveillance [3,4]. Conventional reference methods provide accurate compositional information but are generally destructive, time-consuming, and unsuitable for rapid on-site analysis. NIR spectroscopy enables rapid and non-destructive analysis with minimal sample preparation and has been widely applied to tea quality assessment, including compositional analysis, sensory quality evaluation and geographical-origin discrimination [5,6,7]. Advances in instrument design and manufacturing have made NIR spectrometers more compact, allowing their use beyond the laboratory for incoming material inspection, process monitoring and on-site screening [8,9]. Portable NIR spectrometers have also been used for tea constituent quantification and adulteration screening [10,11].

Although portable NIR spectroscopy is well suited to rapid tea analysis, direct measurement of intact tea leaves remains challenging because their physical heterogeneity can reduce spectral representativeness. Differences in leaf size, shape, orientation and surface structure can produce substantial variation among local measurements [12]. Grinding and homogenisation can reduce this variability, but they require additional sample preparation and reduce the convenience of on-site analysis. An alternative is to improve sampling representativeness without grinding, for example by averaging spectra collected from multiple positions or by increasing the effective sampling area during acquisition. In some benchtop NIR instruments, a rotating sample platform moves different portions of a heterogeneous sample through the measurement field and may therefore provide a more representative spectrum [13]. However, few studies have evaluated this sampling strategy for portable NIR analysis of intact tea leaves.

Efficient and reliable analysis of NIR spectral data is another practical challenge. A typical chemometric workflow involves data ingestion, outlier detection, sample-set partitioning, spectral preprocessing, model development, hyperparameter optimisation and performance evaluation. Scripting environments such as Python and MATLAB offer substantial flexibility for implementing and comparing analytical strategies, but their effective use requires programming expertise, familiarity with chemometric and machine-learning methods, and management of computational environments. Commercial spectroscopy software generally provides user-friendly graphical interfaces and streamlined workflows. However, it can be less flexible for systematic comparisons of preprocessing strategies, model types and datasets from different instruments. This creates a practical tension between analytical flexibility and ease of use for practitioners and researchers without extensive programming experience.

Recent advances in large language models (LLMs) offer a potential way to bridge this gap. Vibe coding has recently emerged as a natural-language-mediated approach to software development, in which users specify objectives and constraints while an LLM generates and iteratively modifies executable code [14,15]. The user’s role therefore shifts from direct code implementation towards problem formulation, code review, result evaluation and iterative feedback. This approach can improve coding efficiency, particularly for researchers with sufficient programming and chemometric expertise to inspect the generated implementation.

Within a vibe-coding workflow, a skill can be defined as a reusable, task-specific package of instructions and supporting resources that guides an LLM in performing a particular class of tasks. By encoding procedural knowledge into reusable workflows, skills can support more consistent and repeatable execution of multi-step analytical tasks [16]. This task-oriented design is well suited to NIR spectral analysis, where data inspection, spectral preprocessing, sample partitioning, model development, optimisation and performance evaluation can be integrated into a coherent analytical workflow. However, to our knowledge, no AI-assisted skill has yet been reported for translating researcher-defined natural-language requirements into executable workflows for NIR spectral analysis.

In the present study, a domain-specific NIR-analysis skill was developed to support the analytical workflow. Using this skill, we evaluated the feasibility of portable NIR spectroscopy for tea product discrimination and moisture prediction using commercial tea samples measured with two portable spectrometers. The ten selected products represented widely consumed green and black teas from different regions of China, while the two instruments enabled comparison of contrasting sampling strategies across complementary spectral ranges. Additional samples from different production batches were used for external validation. Ground and unground samples were compared to further examine how sample state and instrument configuration influenced analytical performance. This study aimed to clarify how sampling configurations influence product discrimination and moisture prediction for heterogeneous tea materials using portable NIR spectrometers, while developing a flexible, AI-assisted, natural-language-driven workflow to facilitate chemometric analysis.

## 2. Materials and Methods

### 2.1. Sample Preparation

Ten commercially available tea products were selected to cover widely consumed green and black teas from different producing regions of China (Table 1). Each product was treated as a separate class for classification modelling. Twenty-four subsamples were randomly taken from each product. Twelve were ground separately and passed through a 40-mesh sieve, whereas the remaining subsamples were retained as unground leaves. For each product and sample state, the twelve subsamples provided eight calibration and four validation samples. The ground and unground subsamples were stored separately in resealable bags in a cool, dry environment until analysis. Before spectral acquisition, the moisture content of each subsample was determined using a halogen moisture analyser (LC-DHS-10A, Zhejiang Lichen Instrument Technology Co., Ltd., Shaoxing, China). The mean moisture contents of the tea products ranged from 1.01% to 5.08%, and no significant difference was observed between the ground and unground states for any tea product (two-sided Student’s *t*-tests, all *p* > 0.05). The prepared samples were then subjected to spectral acquisition as described below.

### 2.2. Spectral Acquisition

For each tea product, spectra were acquired from both ground and unground samples using two portable NIR spectrometers. Both spectrometers were calibrated against a white reference before use. Measurements were conducted at room temperature (approximately 25 °C), and the spectral data used for subsequent analysis were recorded as absorbance.

An NIR-R210 handheld spectrometer (Shenzhen Pynect Science and Technology Co., Ltd., Shenzhen, China) was used to collect spectra over 900–1700 nm. Spectra were acquired separately from the subsamples prepared as described in Section 2.1. For each subsample, spectra were collected at five different positions using six scans per position, and the five position-specific spectra were exported and averaged to obtain one spectral record for modelling.

A NeoSpectra Scanner (Si-Ware Systems, Inc., Menlo Park, CA, USA) was used to collect spectra over 1350–2550 nm. The instrument had a continuously rotating sample platform that allowed different portions of the sample to pass through the measurement field during acquisition. Each subsample was measured separately using an automated sequence of five scans, each lasting 6 s, to obtain a single spectrum for subsequent analysis.

The resulting datasets were denoted as NR-G (NIR-R210, ground), NR-U (NIR-R210, unground), NS-G (NeoSpectra Scanner, ground), and NS-U (NeoSpectra Scanner, unground).

### 2.3. NIR-Analysis Skill for Spectral Data Analysis

In the present study, we developed a domain-specific NIR-analysis skill for spectral analysis in a vibe-coding environment. The skill guides an LLM in translating researcher-defined natural-language requirements into a structured chemometric workflow and provides instructions, methodological references and reusable Python modules for data inspection, spectral preprocessing, dataset partitioning, model development, performance evaluation and visualisation.

Using natural language, researchers can specify the analytical task, data structure, sample categories, target variables, candidate preprocessing methods and calibration models, parameter search ranges, dataset partitioning strategies, evaluation criteria, and required outputs. Before data partitioning, the skill checks source-batch information and class–batch relationships, and reports missing metadata or validation constraints for researcher confirmation. The skill uses the confirmed sample information and validation requirements to determine an appropriate data-partitioning and cross-validation strategy before model development. The skill then converts these requirements into executable analysis steps, performs the requested computations, generates figures and result tables, and records the selected parameters and outputs for review and reporting. Representative task specifications are provided in Supplementary Table S5.

**Figure 1:**
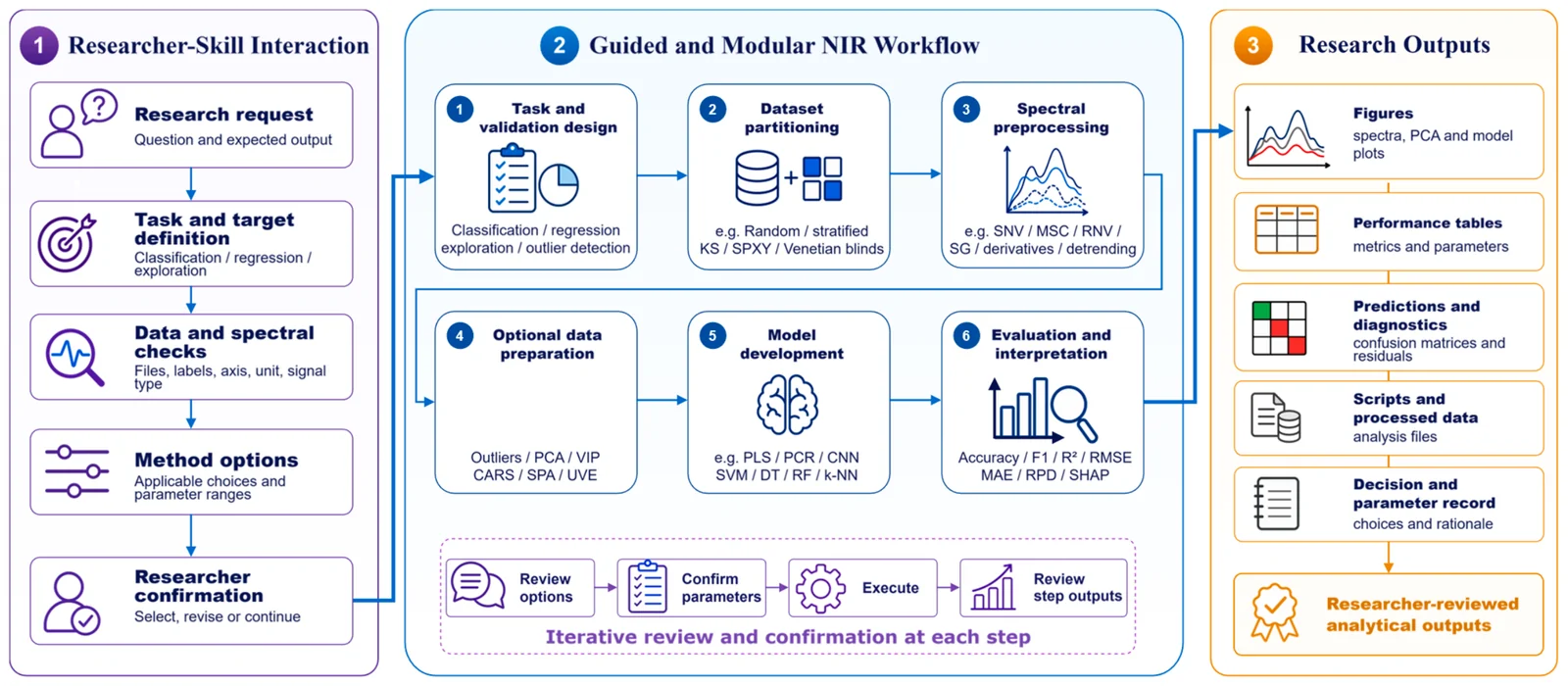
Workflow of the NIR-analysis skill. The workflow comprises task definition and confirmation, six modular analytical steps with iterative researcher review, and generation of analytical outputs.

Figure 1 illustrates the workflow from natural-language task definition to NIR-specific data checks, chemometric analysis, model evaluation and output generation. In the first stage, the researcher defines the task and expected outputs, reviews the data and spectral checks and available method options, and confirms the analysis settings. The second stage comprises six modules: task and validation design, dataset partitioning, spectral preprocessing, optional data preparation, model development, and evaluation and interpretation. The optional data-preparation module can be omitted when it is not required. Researchers can inspect generated code, check data dimensions and parameter settings, and review outputs between modules, revising the analysis as needed. The final stage compiles the analytical outputs.

### 2.4. Spectral Preprocessing and Model Development

All data analysis procedures were conducted within ChatGPT Codex (OpenAI, San Francisco, CA, USA) using the NIR-analysis skill (version 1.1.0) described above. The underlying model version was GPT-5.6 Terra. ChatGPT Codex executed the analytical workflow in Python 3.13.9 (Anaconda), using scikit-learn 1.7.2 for PLS and SVM modelling, NumPy 2.3.5, pandas 2.3.3 and SciPy 1.15.3 for data handling, spectral preprocessing and statistical analyses, and Matplotlib 3.10.6 for plotting. A fixed random seed was used for dataset partitioning and cross-validation to ensure reproducibility.

#### 2.4.1. Dataset Partitioning

The four datasets representing the two instruments and two sample states were analysed separately. For each tea product, eight physically distinct subsamples were randomly assigned to the calibration set and four to the validation set, resulting in 80 calibration records and 40 validation records per dataset (Table 2). The calibration set was used for preprocessing selection and model optimisation, whereas the validation set was reserved for evaluation of the selected models.

#### 2.4.2. Spectral Preprocessing

Sixteen spectral preprocessing conditions were evaluated for each dataset and analytical task. These comprised raw spectra, standard normal variate (SNV), multiplicative scatter correction (MSC), detrending followed by SNV, SNV combined with Savitzky–Golay (SG) smoothing, SNV combined with SG first derivative (D1), SNV combined with SG second derivative (D2), and MSC combined with SG first derivative (D1). For SG smoothing and D1 preprocessing, window lengths of 7, 11 and 15 spectral points were assessed using a second-order polynomial. For D2 preprocessing, window lengths of 11, 15 and 21 spectral points were assessed using a third-order polynomial.

Preprocessing conditions were selected within the calibration set using the cross-validation strategy specified for each analytical task. For transformations requiring parameters estimated from multiple spectra, such as the MSC reference spectrum, these parameters were estimated from the training portion of each cross-validation fold and applied to the corresponding held-out portion. SNV was applied individually to each spectrum. After selection, the preprocessing procedure was fitted using the complete calibration set and applied to the final validation set without further parameter adjustment.

#### 2.4.3. Model Calibration and Optimisation

To compare modelling performance across the two instruments and sample states, partial least squares (PLS) and support vector machine (SVM) were selected to represent linear and nonlinear modelling approaches, respectively. Specifically, PLS discriminant analysis (PLS-DA) and support vector classification (SVC) were used for tea product classification, whereas PLS regression (PLSR) and support vector regression (SVR) were used for moisture prediction. For each subsample, the corresponding tea-product ID was used as the classification label, whereas its measured moisture content was used as the reference value for regression. SVC and SVR were implemented using the radial basis function (RBF) kernel.

For each spectral treatment candidate, PLS-DA and PLSR models were evaluated using 1 to 20 latent variables. Hyperparameters of the SVC and SVR models were optimised by grid search. The regularisation parameter C was tested at 0.001, 0.01, 0.1, 1, 10, 100 and 1000, and the RBF kernel coefficient γ at 10^−6^, 10^−5^, 10^−4^, 10^−3^, 0.01, 0.1 and 1. For SVR, the parameter ε was additionally tested at 0.005, 0.01, 0.02, 0.05, 0.1, 0.2 and 0.3. Different cross-validation strategies were used for classification and moisture prediction. For moisture prediction, preprocessing conditions and model parameters were optimised using leave-one-product-out cross-validation (LOPO-CV) within each calibration set. During each iteration, one tea product with eight calibration spectra was left out for prediction, and the model was fitted using the remaining calibration spectra. For classification, preprocessing conditions and model parameters were optimised using stratified four-fold cross-validation within each calibration set. In each fold, three-quarters of the calibration samples from each tea product were used for model training and the remaining quarter was held out for fold-wise validation, ensuring that all product classes were represented in each training fold. The optimal preprocessing condition and model parameters were selected separately for each model and dataset by maximising cross-validated accuracy for classification and minimising root mean square error of cross-validation for moisture prediction.

#### 2.4.4. Model Validation

A final model using the selected configuration was then fitted to the complete calibration set and evaluated on the corresponding validation set. Classification performance was assessed using accuracy, macro precision, macro recall and macro F1 score, whereas moisture prediction performance was evaluated using the coefficient of determination (R^2^), root mean square error (RMSE), mean absolute error (MAE), bias, standard error of prediction (SEP) and ratio of prediction to deviation (RPD). Macro precision, macro recall and macro F1 were calculated by computing the respective metric for each class and then taking the unweighted mean across classes [17]. Bias was calculated as the mean signed prediction error (predicted minus reference value), and SEP as the standard deviation of the bias-corrected prediction errors [18]. RPD was calculated as the sample standard deviation of the reference values divided by the corresponding RMSE (RMSE_CV_ for cross-validation and RMSEP for validation) [19]. For cross-validation, the corresponding performance metrics are denoted by the subscript CV.

#### 2.4.5. External Validation Design

To further assess the predictive performance of the developed models on independent samples, external validation was conducted. For each tea product, ten additional samples were collected from another production batch of the same manufacturer. Five subsamples were ground separately, while the remaining five were retained as unground leaves. Sample preparation, reference moisture determination, and spectral acquisition followed the procedures described in Section 2.1 and Section 2.2. Details of the external validation samples are summarised in Supplementary Table S1. For each spectrometer and sample state, one spectral record was obtained per sample, resulting in 50 records in each external validation dataset. The previously established preprocessing procedures and trained models were applied without further optimisation or refitting, and their performance was assessed using the same evaluation metrics described above.

#### 2.4.6. Spectral Variability Assessment

To further examine differences in spectral variability under the different sampling configurations, ground and unground samples were compared separately for each spectrometer. Spectra from the calibration set were used for this analysis. Variability within each tea product was quantified using the STD RMS statistic described by Martínez et al. [20]:

(1) $$STD\;RMS=\sqrt{\frac{\sum_{j=1}^{N}\sum_{i=1}^{p}(y_{ij}-{\bar{y}}_{i}{)}^{2}}{p(N-1)}}$$

where $y_{ij}$ is the absorbance at wavelength i in spectrum j, ${\bar{y}}_{i}$ is the mean absorbance at wavelength i across the N spectra, and *p* is the number of spectral wavelength points. A lower STD RMS value indicates lower spectral dispersion among records from the same tea product.

One STD RMS value was calculated for each tea product under each sample state. For each spectrometer, the ten paired product-level STD RMS values for ground and unground samples were compared using a two-sided Wilcoxon signed-rank test. Statistical significance was assessed at *p* < 0.05.

#### 2.4.7. Further Evaluation of the NIR-Analysis Skill

The applicability of the NIR-analysis skill was further examined using three public NIR datasets covering tobacco origin classification and total-sugars prediction, milk fat prediction and paired-instrument corn analysis. Dataset descriptions and detailed results are provided in the Supplementary Materials.

## 3. Results

### 3.1. Raw Absorbance Spectra

**Figure 2:**
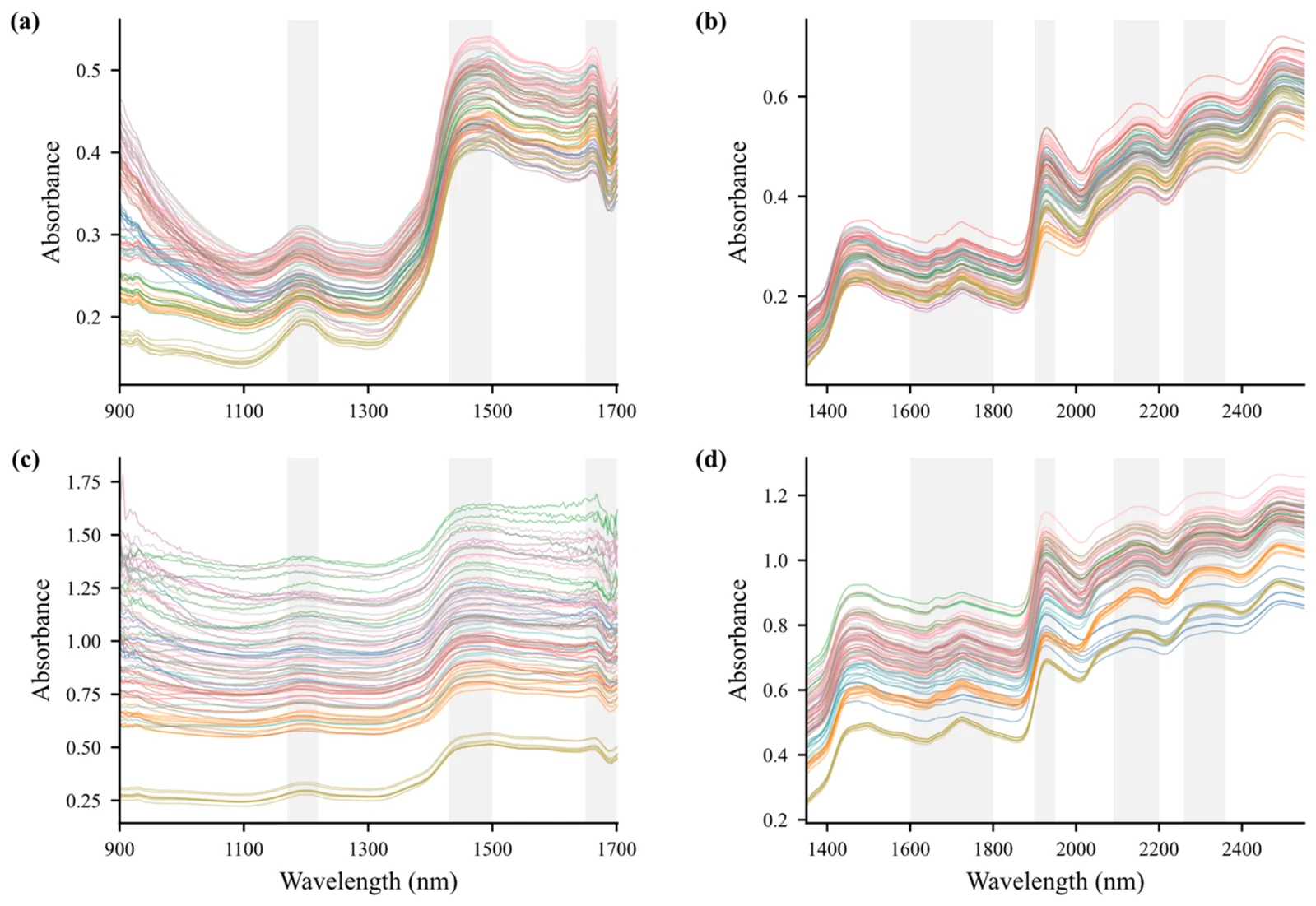
Raw absorbance spectra for (**a**) NR-G, (**b**) NS-G, (**c**) NR-U and (**d**) NS-U. NS, NeoSpectra Scanner; NR, NIR-R210; G, ground; U, unground. Shaded regions indicate the main wavelength ranges discussed in the text. Each line represents the spectrum of an individual sample; colors are used to distinguish the spectra.

The raw absorbance spectra acquired for the four datasets are shown in Figure 2. Within each instrument, the spectra displayed similar overall profiles, although differences in absorbance intensity and local spectral features were observed among tea products and measurement records.

The NIR-R210 spectra (Figure 2a,c) showed weak absorption bands between 1170 and 1220 nm, a broad band between approximately 1430 and 1500 nm, and additional bands between 1650 and 1700 nm, consistent with our previous black tea studies using the same handheld spectrometer [8,10]. The band around 1195 nm may arise mainly from second-overtone C–H and O–H stretching, whereas the band around 1465 nm may be primarily associated with the first overtone of O–H stretching [21]. Absorption around 1650–1680 nm is associated primarily with C–H first overtones and has also been linked to catechin and tea-polyphenol-related spectral information [9,22].

The wider spectral range of the NeoSpectra Scanner revealed additional absorption structure beyond 1700 nm (Figure 2b,d). The strong absorption band around 1900–1950 nm is mainly associated with O–H combination absorption and is therefore strongly influenced by water [23]. In the 1600–1800 nm region, absorption is dominated largely by C–H first overtones; previous tea studies have identified bands around 1648 and 1656–1663 nm as particularly relevant to phenolic compounds and tea polyphenols [22]. At longer wavelengths, several composition-related bands have been reported in tea spectra. Absorption around 2080–2090 nm has been associated with soluble sugars, whereas bands near 2188 nm and 2290 nm contain N–H and C=O related information associated with proteins and free amino acids. The band around 2270 nm involves overlapping C–H and O–H combination absorptions [22]. Bands around 2310 and 2350 nm have been assigned mainly to C–H stretching and bending combinations and have been related to polyphenol- and caffeine-containing tea matrices [24]. However, these band assignments should be interpreted cautiously because overlapping NIR absorption features may reflect contributions from multiple constituents.

For both instruments, the ground samples showed more compact spectral profiles, whereas the unground samples displayed greater between-record variation. This difference was particularly evident for the NIR-R210. The increased variation in unground samples may reflect differences in leaf orientation, particle size and surface structure, together with associated light-scattering effects during reflectance measurement. Comparable effects of sample heterogeneity and measurement procedure have been reported in portable NIR analysis [25,26].

### 3.2. Principal Component Analysis

**Figure 3:**
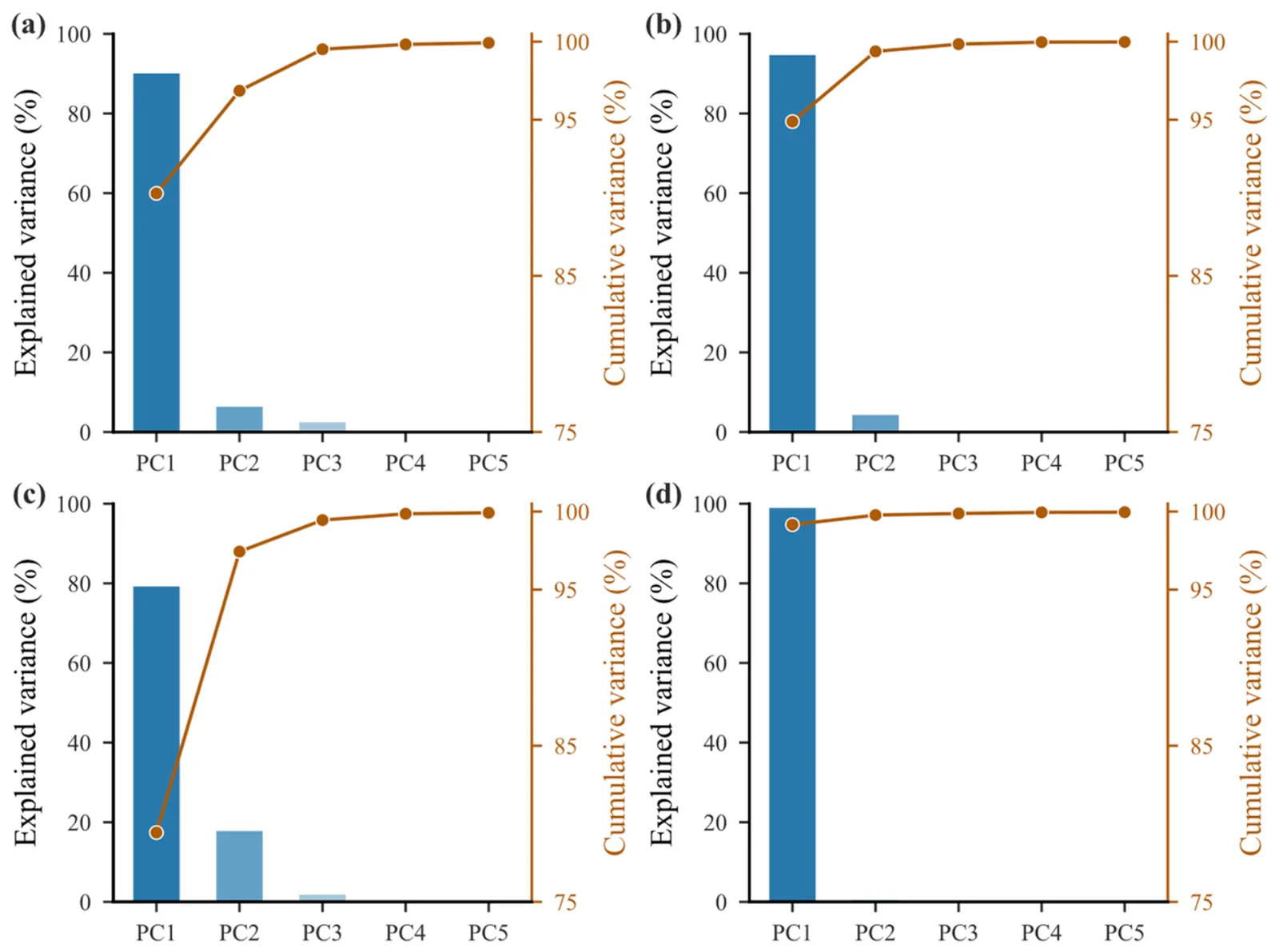
Explained and cumulative variance of the first five principal components for raw calibration spectra from (**a**) NS-G, (**b**) NS-U, (**c**) NR-G and (**d**) NR-U. NS, NeoSpectra Scanner; NR, NIR-R210; G, ground; U, unground. Bars show the variance explained by individual principal components, and lines show the cumulative explained variance.

**Figure 4:**
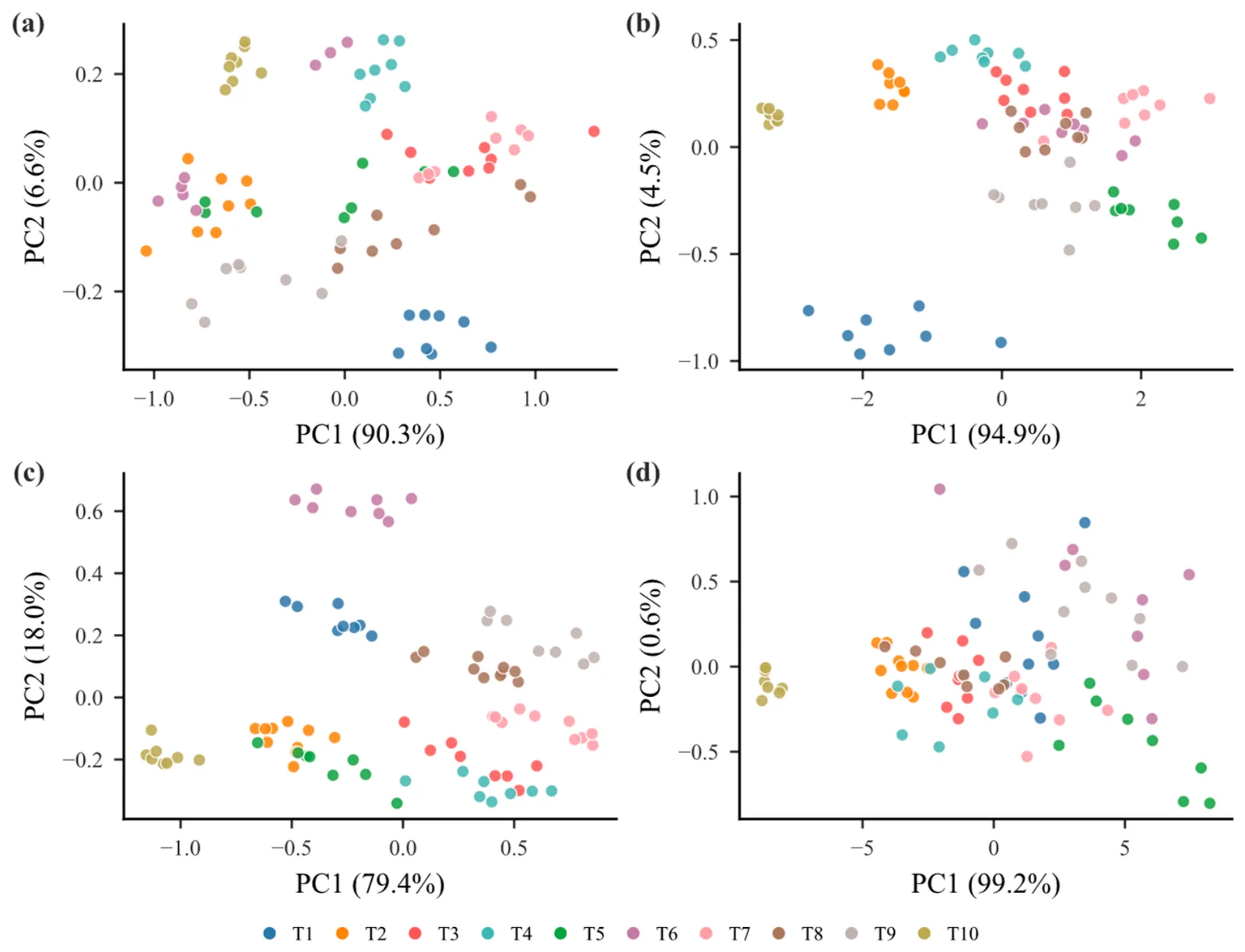
PCA score plots of raw calibration absorbance spectra for (**a**) NS-G, (**b**) NS-U, (**c**) NR-G and (**d**) NR-U. NS, NeoSpectra Scanner; NR, NIR-R210; G, ground; U, unground. Each point represents one subsample, and colours denote the tea products (T1–T10).

Principal component analysis (PCA) was performed separately on the calibration spectra to visualise the overall spectral variation among tea products. The explained variance of the first five principal components is presented in Figure 3, and the corresponding score plots are shown in Figure 4.

For the NeoSpectra Scanner datasets, PC1 and PC2 explained 90.3% and 6.6% of the variance in NS-G (Figure 3a), and 94.9% and 4.5% in NS-U (Figure 3b), respectively. Together, the first two components accounted for 96.9% and 99.4% of the total variance. Both datasets showed product-related clustering in the PC1–PC2 space (Figure 4a,b), although several neighbouring products remained close or partially overlapping. Despite slight differences in the relative contributions of PC1 and PC2, the overall score distributions of NS-G and NS-U were broadly similar.

For the NIR-R210 datasets, PC1 and PC2 explained 79.4% and 18.0% of the spectral variance in NR-G, respectively (Figure 3c). In NR-U, PC1 accounted for 99.2% of the variance, whereas PC2 explained only 0.6% (Figure 3d). The larger contribution of PC2 in NR-G was reflected in a more two-dimensional distribution of the tea products in the score plot (Figure 4c). By contrast, the NR-U scores showed greater overlap among several tea products (Figure 4d). Unground samples showed greater between-record spectral dispersion (Figure 2c,d), which may be associated with variations in leaf orientation, leaf size, surface structure and light scattering. Such variation may have been expressed mainly along PC1, as indicated by the wider PC1 score distributions and higher explained-variance ratios observed in the unground datasets, particularly in NR-U.

### 3.3. Preprocessing and Model Optimisation

Sixteen spectral preprocessing methods were evaluated using PLS and SVM approaches for each dataset, and the optimal model hyperparameters were selected. Table 3 and Table 4 summarise the optimal preprocessing methods and model hyperparameters, together with their cross-validation performance, for the classification and regression models, respectively.

For tea product classification, both PLS-DA and SVC achieved perfect cross-validation performance for the NS-G and NS-U datasets, with all classification metrics equal to 1.000 (Table 3). The NR-G dataset also achieved perfect cross-validation performance with both models, although the selected preprocessing methods differed. Raw spectra were retained for PLS-DA, whereas SNV + SG (D2) was selected for SVC. These perfect scores may reflect distinct product-specific spectral patterns, since the training and held-out portions of cross-validation contained different subsamples of the same commercial products. In contrast, the NR-U dataset was more challenging. The optimal PLS-DA model, based on SNV + SG (D1), obtained an accuracy of 0.800 and a macro F1 score of 0.797, whereas the corresponding SVC model achieved values of 0.838 and 0.839, respectively.

Moisture-prediction performance differed more clearly among the four datasets (Table 4). PLSR yielded higher R^2^_CV_ and lower RMSE_CV_ values than SVR for all four datasets. For NS-G, PLSR and SVR produced similar results, with R^2^_CV_ values of 0.83 and 0.82 and RMSE_CV_ values of 0.569 and 0.579, respectively. For NS-U, PLSR achieved an R^2^_CV_ of 0.84 and an RMSE_CV_ of 0.562, compared with 0.79 and 0.641 for SVR. NR-G showed the best cross-validation performance, with PLSR yielding an R^2^_CV_ of 0.90, an RMSE_CV_ of 0.436 and an RPD_CV_ of 3.174, compared with 0.78, 0.645 and 2.148 for SVR. For NR-U, PLSR achieved an R^2^_CV_ of 0.84, an RMSE_CV_ of 0.566 and an RPD_CV_ of 2.481, compared with 0.81, 0.613 and 2.292 for SVR. Both models selected SNV + SG D1 (7) as the optimal preprocessing method. The selected preprocessing methods varied across instruments, sample states and model types.

### 3.4. Validation of Classification Models

**Figure 5:**
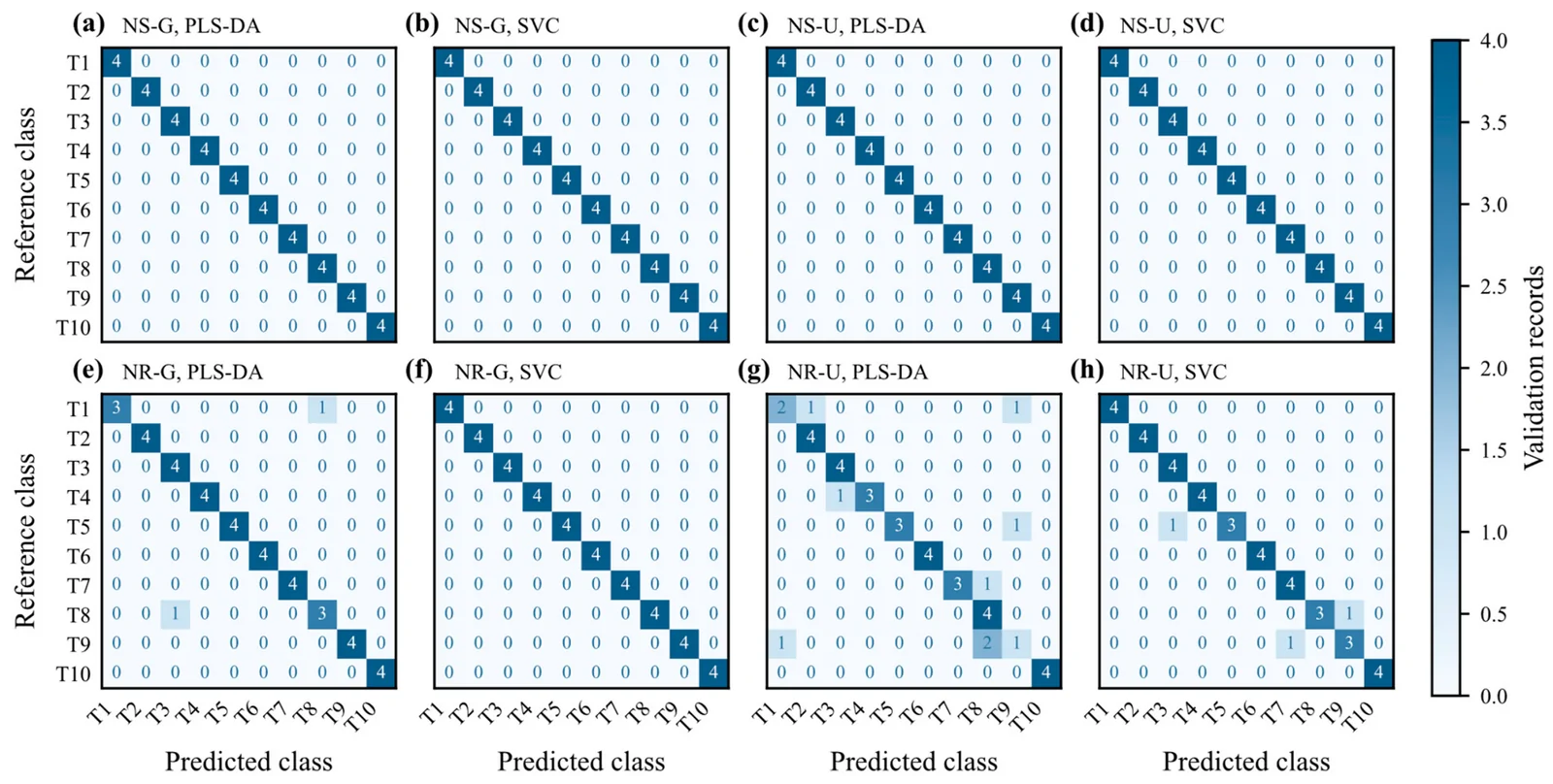
Confusion matrices for the optimised classification models. (**a**,**b**) NS-G, (**c**,**d**) NS-U, (**e**,**f**) NR-G and (**g**,**h**) NR-U. PLS-DA results are shown in (**a**,**c**,**e**,**g**), and SVC results in (**b**,**d**,**f**,**h**). Cell values indicate the numbers of validation records. NS, NeoSpectra Scanner; NR, NIR-R210; G, ground; U, unground.

The validation set was used to evaluate the classification performance of the selected preprocessing methods and models. The classification results are summarised in Table 5, and the corresponding confusion matrices are shown in Figure 5.

For the NeoSpectra Scanner datasets (NS-G and NS-U), both PLS-DA and SVC correctly classified all validation records for the ground and unground samples. Accordingly, accuracy, macro precision, macro recall and macro F1 were all 1.000 for both models (Table 5). The comparable performance for the two sample states suggests that, under the present measurement conditions, both ground and unground samples retained sufficient product-related spectral information for discrimination.

For the NIR-R210 datasets, classification performance differed more clearly between the two sample states. For NR-G, SVC correctly classified all 40 validation records, with all four classification metrics equal to 1.000, whereas PLS-DA misclassified two records and achieved an accuracy and macro F1 score of 0.950 (Figure 5e). For NR-U, the difference between the two models was more apparent. Under their respective optimal preprocessing conditions, SVC achieved an accuracy of 0.925 and a macro F1 score of 0.924 (Figure 5h), whereas PLS-DA yielded an accuracy of 0.800, a macro recall of 0.800, and a macro F1 score of 0.793 (Figure 5g). PLS-DA misclassifications were concentrated in T1 and T9, with two and three errors, respectively, while SVC misclassified one record each from T5, T8 and T9 (Figure 5g,h). Compared with NR-G, accuracy declined less for SVC than for PLS-DA, but both models performed better on ground samples, supporting grinding for more reliable product identification with the NIR-R210. Although spectra from five measurement positions were averaged to obtain one representative record for each sample, substantial spectral variation remained among the intact-leaf samples, likely because leaf orientation, morphology and surface characteristics varied among measurement locations under the point-based sampling approach of the NIR-R210.

### 3.5. Validation of Moisture-Prediction Models

**Figure 6:**
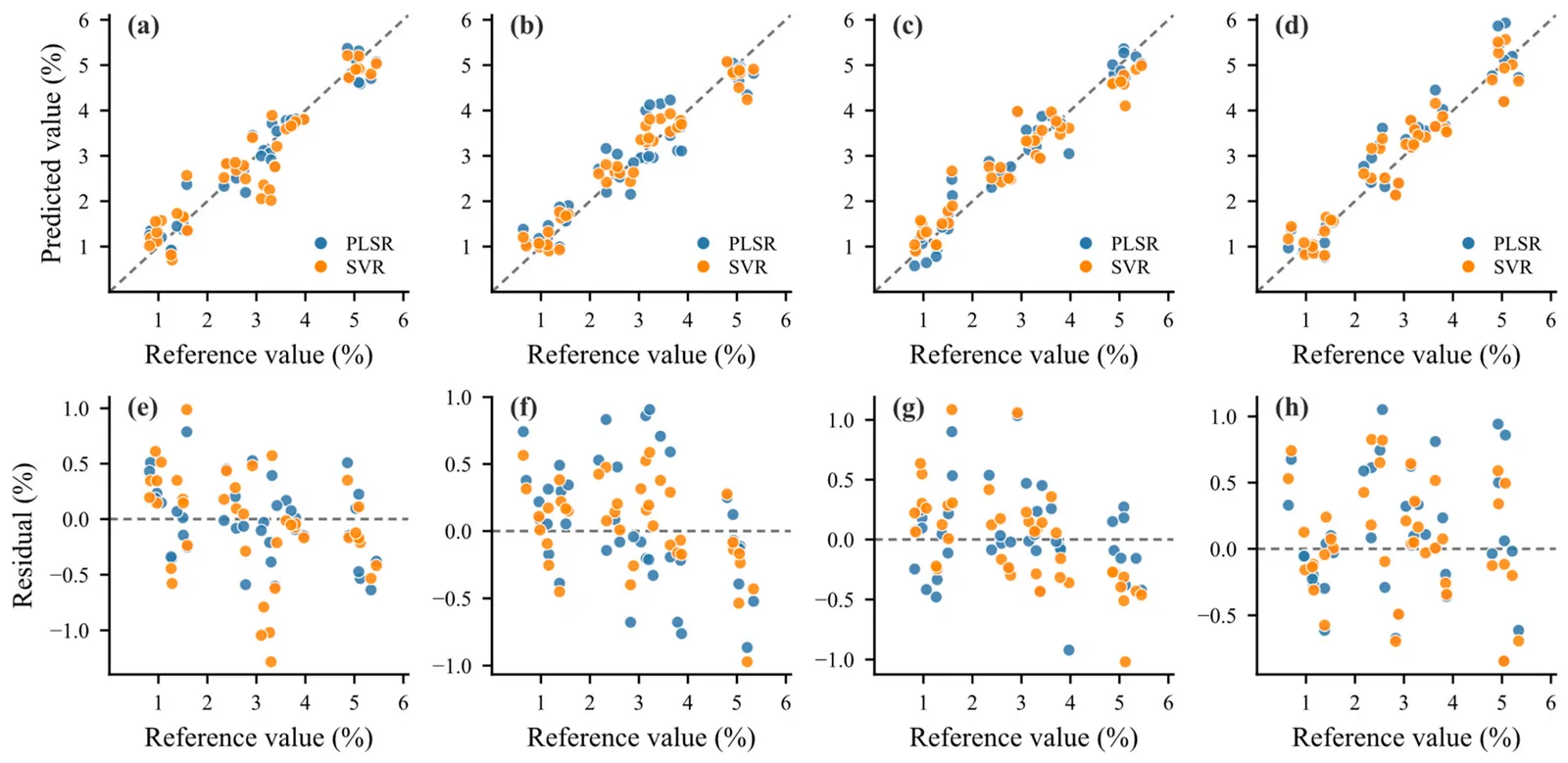
Validation performance of the optimised regression models. (**a**–**d**) Reference versus predicted moisture values for NS-G, NS-U, NR-G and NR-U, respectively. (**e**–**h**) Corresponding residuals plotted against reference moisture values. NS, NeoSpectra Scanner; NR, NIR-R210; G, ground; U, unground.

The validation performance of the optimised PLSR and SVR models for moisture prediction is summarised in Table 6. The relationships between reference and predicted moisture values, together with the residual distributions, are presented in Figure 6.

For the NeoSpectra Scanner datasets, the better-performing model differed between the two sample states. For NS-G, PLSR achieved an R^2^ of 0.94, an RMSEP of 0.340 and an RPD of 4.305, compared with 0.89, 0.480 and 3.046 for SVR. For NS-U, SVR achieved an R^2^ of 0.95, an RMSEP of 0.330 and an RPD of 4.343, compared with 0.90, 0.453 and 3.168 for PLSR, respectively. Both sample states supported good moisture-prediction performance with the NeoSpectra Scanner, and their best-performing models yielded similar prediction errors.

For the NIR-R210 datasets, PLSR outperformed SVR for NR-G. The PLSR model achieved an R^2^ of 0.94, an RMSEP of 0.367 and an RPD of 3.981, compared with 0.92, 0.411 and 3.553 for SVR, respectively. The small bias values obtained for both models further indicate that systematic over- or under-prediction was limited. By contrast, NR-U showed the lowest prediction performance among the four datasets. In this dataset, SVR outperformed PLSR, yielding an R^2^ of 0.91, an RMSEP of 0.423 and an RPD of 3.389; the corresponding PLSR values were 0.89, 0.474 and 3.026. The small difference in RMSEP indicates broadly similar predictive ability for NR-U. Nevertheless, both models performed less well for NR-U than for NR-G, in line with the greater variability observed for unground NIR-R210 spectra.

Across both classification and moisture-prediction tasks, the NeoSpectra Scanner showed comparable performance between ground and unground samples, whereas NR-U was the most challenging dataset. Grinding improved NIR-R210 moisture-prediction performance, while the rotating sample configuration of the NeoSpectra Scanner may have contributed to the performance retained for unground tea samples.

### 3.6. External Validation

External classification results are summarised in Table 7 and Supplementary Figure S1. Both PLS-DA and SVC correctly classified all 50 external records in each NeoSpectra Scanner dataset. For NR-G, PLS-DA and SVC achieved accuracies of 0.960 and 1.000, respectively. For NR-U, the corresponding accuracies were 0.640 and 0.800, below the original validation values of 0.800 and 0.925. The lower external accuracies for NR-U indicate that product identification from intact leaves was less reliable in the additional production batch, highlighting the practical advantage of grinding for NIR-R210 measurements.

External validation results for moisture prediction are summarised in Table 8 and Supplementary Figure S2. For NS-G, PLSR achieved an R^2^ of 0.91 and an RMSEP of 0.422, compared with 0.88 and 0.481 for SVR, respectively. For NS-U, SVR performed better (R^2^ = 0.89, RMSEP = 0.462) than PLSR (R^2^ = 0.84, RMSEP = 0.565). For NR-G, PLSR yielded an R^2^ of 0.86 and an RMSEP of 0.529, compared with 0.82 and 0.603 for SVR, respectively. NR-U showed the largest prediction errors, with SVR achieving an R^2^ of 0.68 and an RMSEP of 0.797, compared with 0.57 and 0.922 for PLSR, respectively. RMSEP values for the external validation samples were higher than those for the original validation samples across all moisture-prediction models, with the largest increases observed for NR-U. Nevertheless, the relative performance of PLSR and SVR remained consistent. PLSR produced lower prediction errors for ground samples, whereas SVR performed better for unground samples with both spectrometers.

### 3.7. Spectral Variability

**Figure 7:**
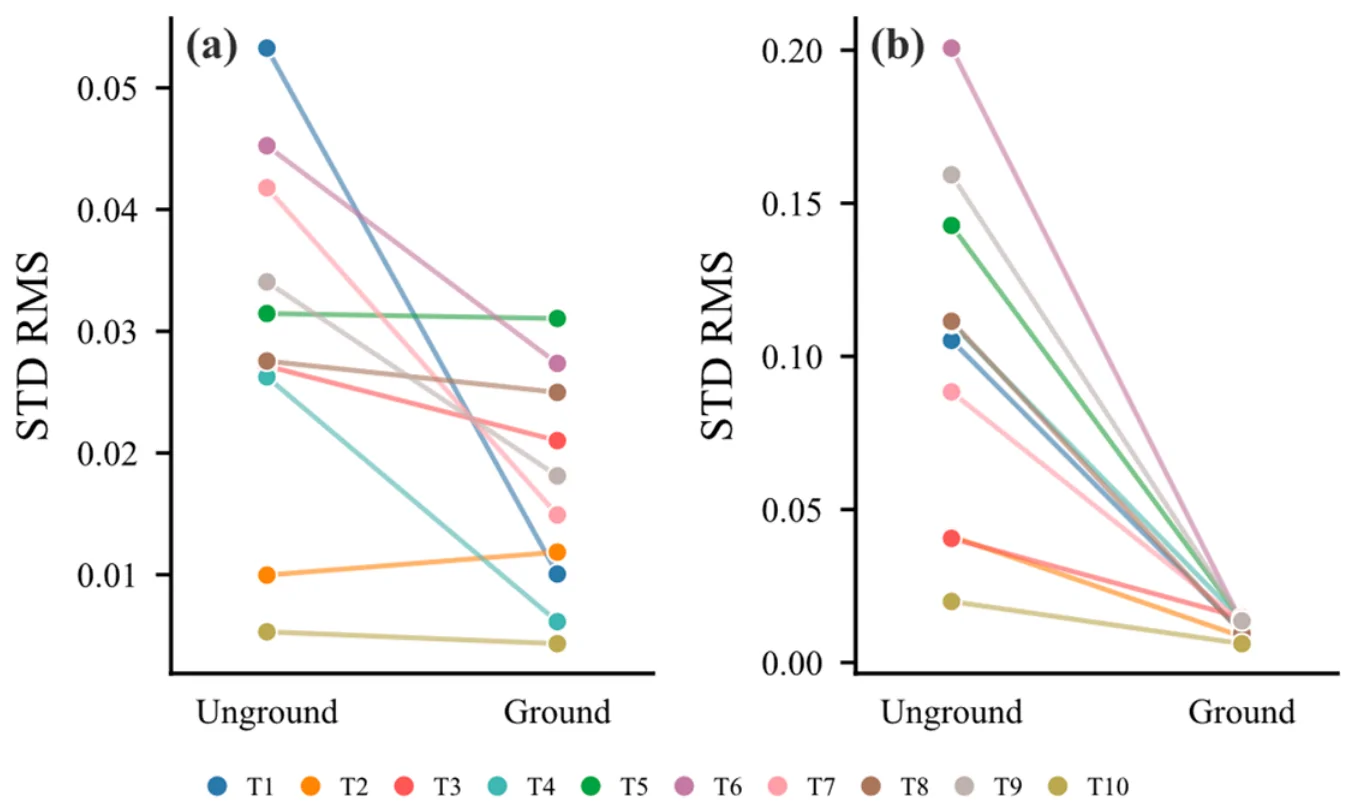
Paired STD RMS values for unground and ground samples. (**a**) NeoSpectra Scanner; (**b**) NIR-R210. Lines connect values from the same tea product.

Spectral variability within each tea product was quantified using the STD RMS statistic based on the raw calibration absorbance spectra. The product-level values are shown as paired observations in Figure 7, and the median STD RMS values and paired Wilcoxon signed-rank test results are summarised in Table 9.

For the NeoSpectra Scanner, grinding reduced spectral variability (Figure 7a). The median STD RMS decreased from 0.030 for unground samples to 0.017 for ground samples (*p* = 0.010). Nine of the ten tea products showed lower STD RMS values after grinding, whereas T2 showed a small increase from 0.0100 to 0.0119. For the NIR-R210 datasets, grinding also reduced the product-level STD RMS values for all ten tea products (Figure 7b). The median STD RMS decreased from 0.108 for unground samples to 0.013 for ground samples, and the paired difference was significant (*p* = 0.002).

The ground–unground difference in spectral variability was greater for the NIR-R210 than for the NeoSpectra Scanner. This pattern may partly be related to the rotating sampling configuration of the NeoSpectra Scanner, which allowed different portions of the heterogeneous sample to pass through the measurement field during acquisition and may have reduced the influence of local sample heterogeneity on the recorded spectra.

## 4. Discussion

Instrument configuration and sample state were closely associated with classification and moisture-prediction performance. The NeoSpectra Scanner produced accurate tea product classification and moisture prediction for both ground and unground tea samples. The NIR-R210 was more sensitive to sample state. Grinding produced more consistent spectra and supported more accurate classification and moisture prediction, whereas the unground NIR-R210 samples showed the lowest performance for both tasks. The difference in spectral variability between ground and unground samples was also more evident for the NIR-R210 than for the NeoSpectra Scanner. Previous studies have reported that rotating sampling configurations can improve spectral repeatability and analytical performance for heterogeneous materials [13,26]. The rotating platform of the NeoSpectra Scanner may similarly have improved sampling representativeness by reducing the influence of local differences in leaf orientation, size, shape and surface structure. However, this potential contribution requires further validation.

In moisture prediction, PLSR performed better for NS-G and NR-G in the validation sets, whereas SVR performed better for NS-U and NR-U. The greater spectral variability of unground samples may have made the relationship between spectra and moisture content more complex and nonlinear, favouring SVR. Previous tea studies have reported advantages of SVR [27,28,29], whereas the better model here depended on sample state. Moreover, high prediction accuracy with both benchtop and portable instruments has mainly been reported for homogeneous tea powders [6,9], while accuracy decreased from powder to intact leaves [22]. Consistent with these findings, the NIR-R210 achieved higher accuracy for ground samples, whereas performance decreased for unground samples and further declined in external validation, indicating that the applicability of portable instruments to intact finished teas remains limited.

For classification, SVC outperformed PLS-DA for the two NIR-R210 datasets, while both models performed equally well with the NeoSpectra Scanner. Previous portable NIR studies have also reported advantages of nonlinear classification models, including our previous studies on black tea adulteration and colourant detection [8,10] and a study of milk authenticity [30]. These observations indicate that nonlinear models can offer advantages for portable NIR analysis when the spectral relationships are complex, although their relative performance depends on the characteristics of the dataset and analytical task.

The NeoSpectra Scanner showed potential for rapid analysis of both ground and unground commercial tea samples, while the NIR-R210 performed more reliably with ground samples. External validation showed broadly similar performance patterns across instruments and sample states, although moisture-prediction errors increased for all models and classification accuracy declined markedly for NR-U. The greater sensitivity of moisture prediction may reflect the distinct analytical requirements of classification and regression. Product discrimination depends on sufficient separation between class-related spectral patterns, whereas accurate moisture prediction requires more precise extraction of water-related information from spectral responses affected by sample matrix and scattering variations. Consequently, spectral variations that do not alter class assignments may still introduce appreciable errors in quantitative prediction. A similar distinction between discriminative and quantitative capabilities was observed in our previous portable NIR study of colourants in black tea [10]. The concurrent decline in classification and moisture-prediction performance for NR-U further highlights the importance of spectral representativeness. For point-based measurements of intact leaves, increasing the number or spatial distribution of measurement positions may improve analytical robustness, although the additional acquisition time should be considered in practical applications.

The NIR-analysis skill developed in this study provided a domain-specific workflow that converted researcher-defined natural-language requirements into spectral inspection, preprocessing, model calibration, validation and visualisation. Compared with a conventional manually scripted workflow, the skill reduced the coding required for routine analysis through reusable modules; however, LLM-generated code may still contain errors, so researcher inspection of the generated code and intermediate outputs remains necessary. The applicability of the workflow was further examined using public NIR datasets for tobacco origin classification and total-sugars prediction, milk fat prediction and paired-instrument corn analysis, with the corresponding results summarised in the Supplementary Materials.

The present tea study included only ten commercial tea products, and external validation used another production batch of the same products from the same manufacturers. The results therefore support product discrimination and moisture prediction within the investigated product and batch range, but do not establish comparable performance for new tea products, other tea types or geographical origins, products from previously unseen manufacturers, or a wider range of moisture contents. Further evaluation across production batches, seasons and instruments is needed to assess the broader applicability of the models and workflow.

## 5. Conclusions

This study demonstrated the feasibility of portable NIR spectroscopy for rapid tea product discrimination and moisture prediction. We measured and compared ground and unground tea samples with two portable spectrometers, with a domain-specific NIR-analysis skill supporting the analytical workflow. Analytical performance varied with instrument configuration, sample state and modelling approach. The validation results showed that the NeoSpectra Scanner achieved 100% classification accuracy for both sample states and showed good moisture-prediction performance. The NIR-R210 also performed strongly after grinding, whereas unground leaves were more challenging for both classification and moisture prediction. External validation showed similar performance patterns across instruments and sample states, although moisture-prediction errors increased and classification accuracy declined for unground NIR-R210 samples. However, the observed performance does not necessarily indicate comparable performance on new tea products or products from different manufacturers or geographical origins.

Grinding significantly reduced spectral variability, particularly for the NIR-R210. The performance obtained with unground samples may be associated with the rotating sampling configuration of the NeoSpectra Scanner.

These findings highlight the importance of representative sampling and appropriate sample presentation in portable NIR analysis of heterogeneous tea materials. The NIR-analysis skill provided a flexible workflow to support the analytical tasks in this study. Its applicability was preliminarily examined using public NIR datasets covering classification, quantitative prediction and paired-instrument analysis. Further evaluation across a wider range of tea types, production batches, seasons and instruments is needed to assess the broader applicability of the portable NIR approach and analytical workflow.

## Figures and Tables

**Table 1:** Tea products and reference moisture contents. Values are means ± standard deviations for each tea product.

ID	Tea Type	Product	Origin	Moisture (%)
T1	Black tea	Jingye Hongcha	Changning, Yunnan	5.01 ± 0.15
T2	Green tea	Xinong Chunlv	Ya’an, Sichuan	1.01 ± 0.17
T3	Green tea	Maofeng	Yongchuan, Chongqing	1.56 ± 0.11
T4	Green tea	Maofeng	Wanzhou, Chongqing	1.03 ± 0.16
T5	Green tea	Daye Lvcha	Lincang, Yunnan	2.50 ± 0.29
T6	Black tea	Daye Hongcha	Changning, Yunnan	2.71 ± 0.24
T7	Green tea	Yongchuan Xiuya	Yongchuan, Chongqing	3.77 ± 0.12
T8	Black tea	Shengshou Yunhua	Dazu, Chongqing	5.08 ± 0.15
T9	Black tea	Fohai Hongcha	Menghai, Yunnan	3.35 ± 0.15
T10	Green tea	Enshi Yulu	Enshi, Hubei	3.25 ± 0.11

**Table 2:** Composition and partitioning of the modelling datasets.

Dataset	Instrument	Sample State	Total Records	Calibration Set	Validation Set
NS-G	NeoSpectra Scanner	Ground	120	80	40
NS-U	NeoSpectra Scanner	Unground	120	80	40
NR-G	NIR-R210	Ground	120	80	40
NR-U	NIR-R210	Unground	120	80	40

**Table 3:** Cross-validation performance of the optimal spectral preprocessing for classification.

Dataset	Model	Preprocessing	Parameters	Accuracy	Macro Precision	Macro Recall	Macro F1
NS-G	PLS-DA	Raw	LV = 10	1.000	1.000	1.000	1.000
NS-G	SVC	SNV	C = 1; γ = 0.01	1.000	1.000	1.000	1.000
NS-U	PLS-DA	Raw	LV = 12	1.000	1.000	1.000	1.000
NS-U	SVC	SNV + SG D2 (11)	C = 0.001; γ = 1 × 10^−6^	1.000	1.000	1.000	1.000
NR-G	PLS-DA	Raw	LV = 12	1.000	1.000	1.000	1.000
NR-G	SVC	SNV + SG D2 (11)	C = 0.001; γ = 1 × 10^−6^	1.000	1.000	1.000	1.000
NR-U	PLS-DA	SNV + SG D1 (15)	LV = 15	0.800	0.816	0.800	0.797
NR-U	SVC	SNV + SG D1 (11)	C = 1000; γ = 1 × 10^−5^	0.838	0.843	0.838	0.839

Numbers in parentheses indicate SG window lengths in spectral points. D1 and D2 denote first and second derivatives. LV, number of latent variables; C and γ, SVC hyperparameters. NS, NeoSpectra Scanner; NR, NIR-R210; G, ground; U, unground.

**Table 4:** Cross-validation performance of the optimal spectral preprocessing for regression.

Dataset	Model	Preprocessing	Parameters	R^2^_CV_	RMSE_CV_ (%)	MAE_CV_ (%)	Bias_CV_ (%)	SEP_CV_ (%)	RPD_CV_
NS-G	PLSR	Detrend + SNV	LV = 6	0.83	0.569	0.478	−0.108	0.559	2.433
NS-G	SVR	Raw	C = 1000; γ = 0.0001; ε = 0.1	0.82	0.579	0.442	−0.047	0.577	2.391
NS-U	PLSR	Raw	LV = 3	0.84	0.562	0.443	−0.090	0.555	2.502
NS-U	SVR	Raw	C = 1000; γ = 0.0001; ε = 0.2	0.79	0.641	0.457	−0.207	0.606	2.193
NR-G	PLSR	Raw	LV = 7	0.90	0.436	0.359	0.009	0.436	3.174
NR-G	SVR	SNV + SG D1 (11)	C = 10; γ = 0.0001; ε = 0.2	0.78	0.645	0.530	−0.106	0.636	2.148
NR-U	PLSR	SNV + SG D1 (7)	LV = 11	0.84	0.566	0.454	−0.074	0.561	2.481
NR-U	SVR	SNV + SG D1 (7)	C = 1000; γ = 1 × 10^−5^; ε = 0.1	0.81	0.613	0.488	−0.031	0.612	2.292

Numbers in parentheses indicate SG window lengths in spectral points. D1 and D2 denote first and second derivatives. LV, number of latent variables; C, γ and ε, SVR hyperparameters. NS, NeoSpectra Scanner; NR, NIR-R210; G, ground; U, unground.

**Table 5:** Validation performance of the optimised classification models.

Dataset	Model	Accuracy	Macro Precision	Macro Recall	Macro F1
NS-G	PLS-DA	1.000	1.000	1.000	1.000
NS-G	SVC	1.000	1.000	1.000	1.000
NS-U	PLS-DA	1.000	1.000	1.000	1.000
NS-U	SVC	1.000	1.000	1.000	1.000
NR-G	PLS-DA	0.950	0.955	0.950	0.950
NR-G	SVC	1.000	1.000	1.000	1.000
NR-U	PLS-DA	0.800	0.817	0.800	0.793
NR-U	SVC	0.925	0.935	0.925	0.924

NS, NeoSpectra Scanner; NR, NIR-R210; G, ground; U, unground.

**Table 6:** Validation performance of the optimised regression models.

Dataset	Model	R^2^	RMSEP (%)	MAE (%)	Bias (%)	SEP (%)	RPD
NS-G	PLSR	0.94	0.340	0.273	−0.007	0.340	4.305
NS-G	SVR	0.89	0.480	0.372	−0.053	0.477	3.046
NS-U	PLSR	0.90	0.453	0.365	0.052	0.450	3.168
NS-U	SVR	0.95	0.330	0.267	0.041	0.328	4.343
NR-G	PLSR	0.94	0.367	0.269	0.028	0.366	3.981
NR-G	SVR	0.92	0.411	0.327	0.008	0.411	3.553
NR-U	PLSR	0.89	0.474	0.369	0.105	0.462	3.026
NR-U	SVR	0.91	0.423	0.333	0.072	0.417	3.389

NS, NeoSpectra Scanner; NR, NIR-R210; G, ground; U, unground.

**Table 7:** External validation performance of the optimised classification models.

Dataset	Model	Accuracy	Macro Precision	Macro Recall	Macro F1
NS-G	PLS-DA	1.000	1.000	1.000	1.000
NS-G	SVC	1.000	1.000	1.000	1.000
NS-U	PLS-DA	1.000	1.000	1.000	1.000
NS-U	SVC	1.000	1.000	1.000	1.000
NR-G	PLS-DA	0.960	0.967	0.960	0.960
NR-G	SVC	1.000	1.000	1.000	1.000
NR-U	PLS-DA	0.640	0.665	0.640	0.633
NR-U	SVC	0.800	0.850	0.800	0.790

NS, NeoSpectra Scanner; NR, NIR-R210; G, ground; U, unground.

**Table 8:** External validation performance of the optimised regression models.

Dataset	Model	R^2^	RMSEP (%)	MAE (%)	Bias (%)	SEP (%)	RPD
NS-G	PLSR	0.91	0.422	0.307	0.039	0.420	3.367
NS-G	SVR	0.88	0.481	0.366	0.032	0.480	2.953
NS-U	PLSR	0.84	0.565	0.419	0.077	0.560	2.495
NS-U	SVR	0.89	0.462	0.320	0.047	0.459	3.053
NR-G	PLSR	0.86	0.529	0.405	−0.039	0.528	2.683
NR-G	SVR	0.82	0.603	0.466	−0.068	0.599	2.355
NR-U	PLSR	0.57	0.922	0.605	0.038	0.922	1.533
NR-U	SVR	0.68	0.797	0.527	0.021	0.796	1.774

NS, NeoSpectra Scanner; NR, NIR-R210; G, ground; U, unground.

**Table 9:** Median STD RMS values for ground and unground tea samples.

Instrument	Unground Median STD RMS	Ground Median STD RMS	*p*
NIR-R210	0.108	0.013	0.002
NeoSpectra Scanner	0.030	0.017	0.010

Values are medians of the ten product-level STD RMS values. *p* values are from two-sided paired Wilcoxon signed-rank tests.

## Data Availability

The data presented in this study are not readily available because they form part of an ongoing research project and are being used for further analyses. The data are available from the corresponding author upon reasonable request. The NIR-analysis skill (version 1.1.0) used in this study is publicly available as the tagged release v1.1.0 on GitHub: https://github.com/chenjia5268/nir-analysis-skill/releases/tag/v1.1.0 (accessed on 25 October 2026).

## Supplementary Materials

The following supporting information can be downloaded at: https://www.mdpi.com/article/10.3390/foods15213855/s1, Table S1: External validation sample information; Table S2: Validation performance for tobacco-origin classification and total-sugars prediction; Table S3: Validation performance for milk-fat prediction; Table S4: Moisture-prediction performance for paired corn samples measured on three instruments; Table S5: Representative natural-language task specifications; Figure S1: Confusion matrices of the external validations; Figure S2: External validation performance of the optimised regression models; Figure S3: Validation results for tobacco; Figure S4: Validation of milk-fat prediction; Figure S5: Validation results for corn moisture.

## Abbreviations

The following abbreviations are used in this manuscript:

CV	cross-validation
D1	first derivative
D2	second derivative
F1	F1 score
LLM	large language model
LOPO-CV	leave-one-product-out cross-validation
LV	latent variable
MAE	mean absolute error
MSC	multiplicative scatter correction
NIR	near-infrared
NR-G	NIR-R210, ground
NR-U	NIR-R210, unground
NS-G	NeoSpectra Scanner, ground
NS-U	NeoSpectra Scanner, unground
PCA	principal component analysis
PLS	partial least squares
PLS-DA	partial least squares discriminant analysis
PLSR	partial least squares regression
R^2^	coefficient of determination
RBF	radial basis function
RMSE	root mean square error
RMSE_CV_	root mean square error of cross-validation
RMSEP	root mean square error of prediction
RPD	ratio of prediction to deviation
SEP	standard error of prediction
SG	Savitzky–Golay
SNV	standard normal variate
STD RMS	standard-deviation root mean square
SVC	support vector classification
SVM	support vector machine
SVR	support vector regression
