# Supplementary material for: An AI-Assisted Workflow for Tea Product Discrimination and Moisture Prediction Using Portable NIR Spectroscopy
Source: Foods. 2026 Oct 28;15(21):3855. doi: 10.3390/foods15213855 (PMC13763540; doi:10.3390/foods15213855)
Supplement: Supplementary file 1 [file foods-15-03855-s001.zip › foods-4637702-supplementary.pdf]

Supplementary Materials

An AI-Assisted Workflow for Tea Product Discrimination and Moisture Prediction Using Portable NIR Spectroscopy

Zhengfei Luo [1,2,†], Bo Zhao [3,†], Qing Meng [4,*], Dongling Qiao [1], Yanhong Liu [2], Guohua Zhao [5] and Jia Chen [1,*]

[1] Chinese-Hungarian Cooperative Research Centre for Food Science, College of Food Science, Southwest University, Chongqing 400715, China; qdttkl@163.com (D.Q.)
[2] Yunnan Key Laboratory of Tea Germplasm Conservation and Utilization in the Lancang River Basin, College of Biotechnology and Engineering, West Yunnan University, Lincang 677000, China; luopyh@126.com (Z.L.); Liu41294777@126.com (Y.L.)
[3] Key Laboratory of Condiment Supervision Technology, State Administration for Market Regulation, Chongqing Institute for Food and Drug Control, Chongqing 400731, China; zhaobocq@163.com (B.Z.)
[4] Integrative Science Center of Germplasm Creation in Western China (Chongqing) Science City, College of Food Science, Southwest University, Chongqing 400715, China
[5] College of Life Sciences, Sichuan Normal University, Chengdu 610101, China; zhaogh@sicnu.edu.cn (G.Z.)
* Correspondence: mengqing2015@swu.edu.cn (Q.M.); swaumyh@swu.edu.cn (J.C.)
† These authors contributed equally to this work.

Table S1. External validation sample information. Values are means ± standard deviations for each tea product.

ID	Tea type	Product	Origin	Number of samples (n)	Moisture (%)
T1	Black tea	Jingye Hongcha	Changning, Yunnan	10	4.73 ± 0.86
T2	Green tea	Xinong Chunlv	Ya'an, Sichuan	10	1.05 ± 0.11
T3	Green tea	Maofeng	Yongchuan, Chongqing	10	1.56 ± 0.31
T4	Green tea	Maofeng	Wanzhou, Chongqing	10	1.14 ± 0.22
T5	Green tea	Daye Lvcha	Lincang, Yunnan	10	2.36 ± 0.41
T6	Black tea	Daye Hongcha	Changning, Yunnan	10	2.88 ± 0.65
T7	Green tea	Yongchuan Xiuya	Yongchuan, Chongqing	10	3.61 ± 0.46
T8	Black tea	Shengshou Yunhua	Dazu, Chongqing	10	4.96 ± 0.70
T9	Black tea	Fohai Hongcha	Menghai, Yunnan	10	3.34 ± 0.59
T10	Green tea	Enshi Yulu	Enshi, Hubei	10	3.29 ± 0.42

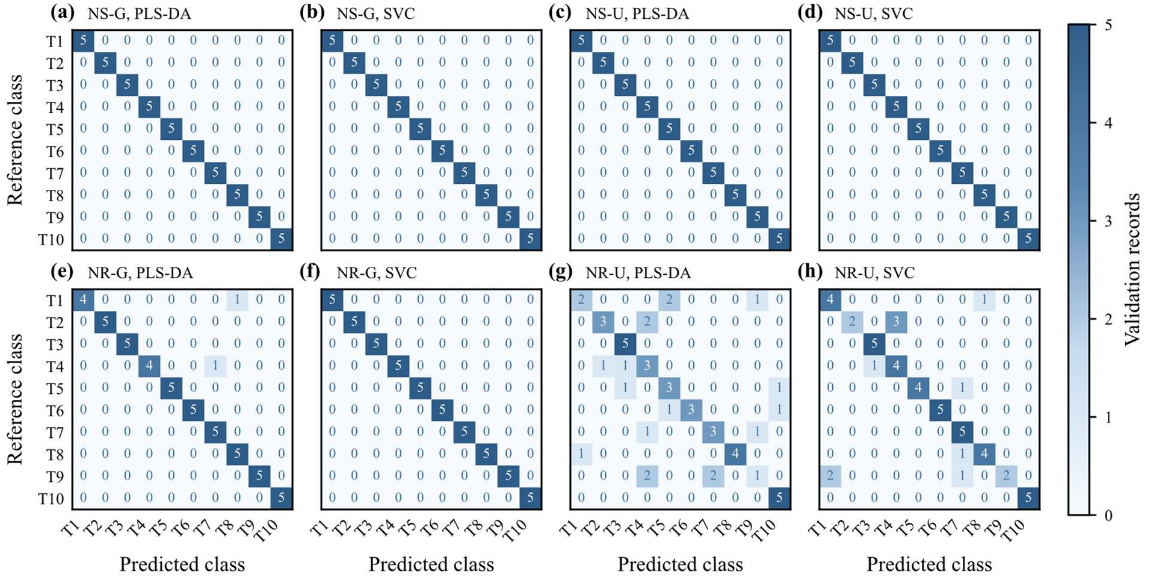

Figure S1. Confusion matrices of the external validations. Cell values indicate the numbers of validation records. NS, NeoSpectra Scanner; NR, NIR-R210; G, ground; U, unground.

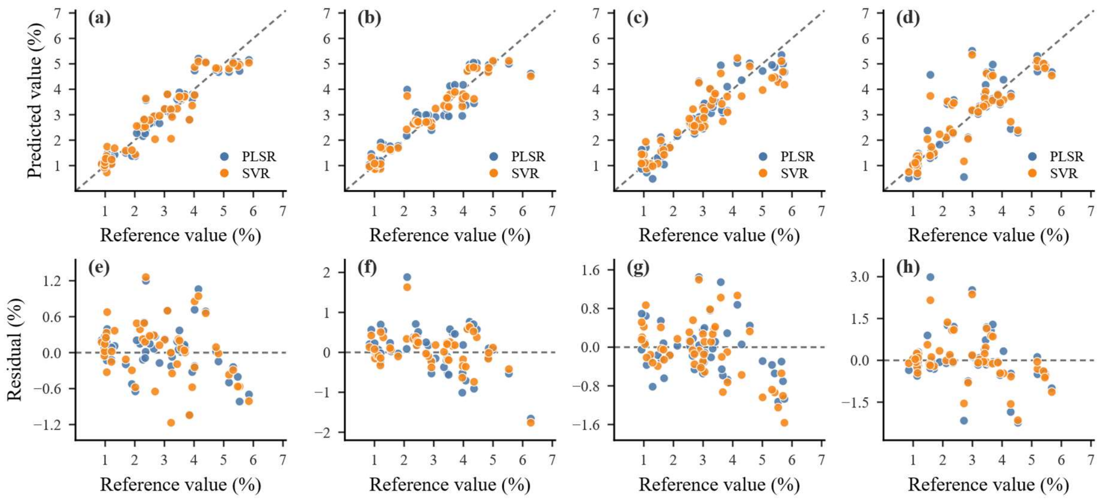

Figure S2. External validation performance of the optimised regression models. (a–d) Reference versus predicted moisture values for NS-G, NS-U, NR-G and NR-U, respectively. (e–h) Corresponding residuals plotted against reference moisture values. NS, NeoSpectra Scanner; NR, NIR-R210; G, ground; U, unground.

Tobacco: origin classification and total-sugars prediction

The tobacco dataset contained 347 absorbance spectra linked one-to-one with country-of-origin labels and chemical reference measurements. Tanzania (n = 2) was excluded from origin classification because a stratified validation partition was not feasible. The remaining 345 spectra were split into 276 calibration and 69 validation spectra. Total-sugars regression used all 347 spectra, with 277 calibration and 70 validation spectra.

Dataset download address: https://data.mendeley.com/datasets/9z7dgdtggk/1

Table S2. Validation performance for tobacco-origin classification and total-sugars prediction.

Tasks	Model	Selected workflow	Performance
Classification	PLS-DA	PLS-DA/SNV/LV=4	Accuracy 0.783; balanced accuracy 0.516; macro F1 0.503
	SVC	SVC/SNV/C=10 RBF; γ = 'scale'	Accuracy 0.899; balanced accuracy 0.644; macro F1 0.663
Regression	PLSR	PLSR/SNV/LV=12	R^2 0.947; RMSEP 0.942; RPD 4.39
	SVR	SVR/SNV/C=10 RBF; γ = 'scale'; ε = 0.05	R^2 0.914; RMSEP 1.207; RPD 3.43

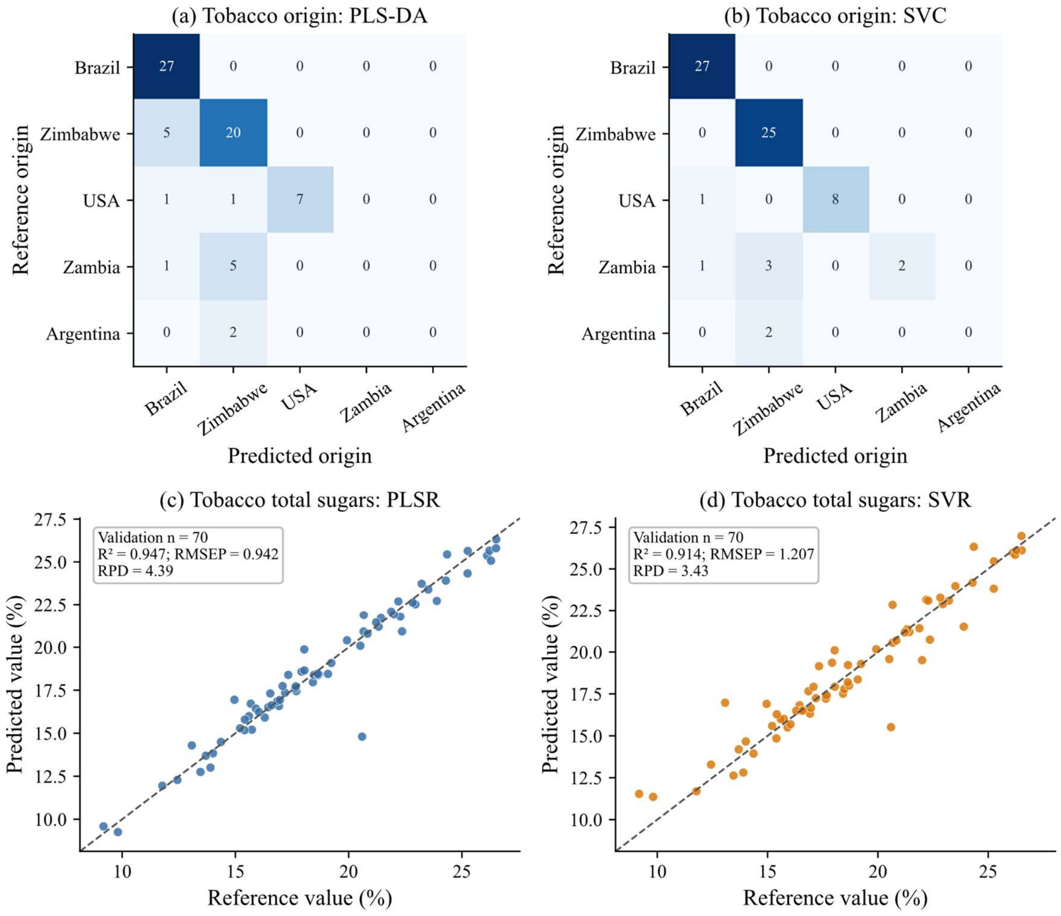

Figure S3. Validation results for tobacco. (a) PLS-DA and (b) SVC origin classification; (c) PLSR and (d) SVR total-sugars prediction.

Milk: prediction of fat content

The milk dataset comprised 1224 spectral records from 41 cows. Data from 32 cows (970 records) were used for calibration, while data from the remaining 9 cows (254 records) were used for validation.

Dataset download address: https://zenodo.org/records/8263430/files/data_table.csv?download=1

Table S3. Validation performance for milk-fat prediction.

Model	Selected workflow	Performance
PLSR	PLSR/Raw/LV=8	R^2 0.913; RMSEP 0.240; RPD 3.39
SVR	SVR/SNV+SG/C=10<br>SG: 11 points, order 2; derivative 0<br>RBF; γ = 'scale'; ε = 0.05	R^2 0.893; RMSEP 0.266; RPD 3.07

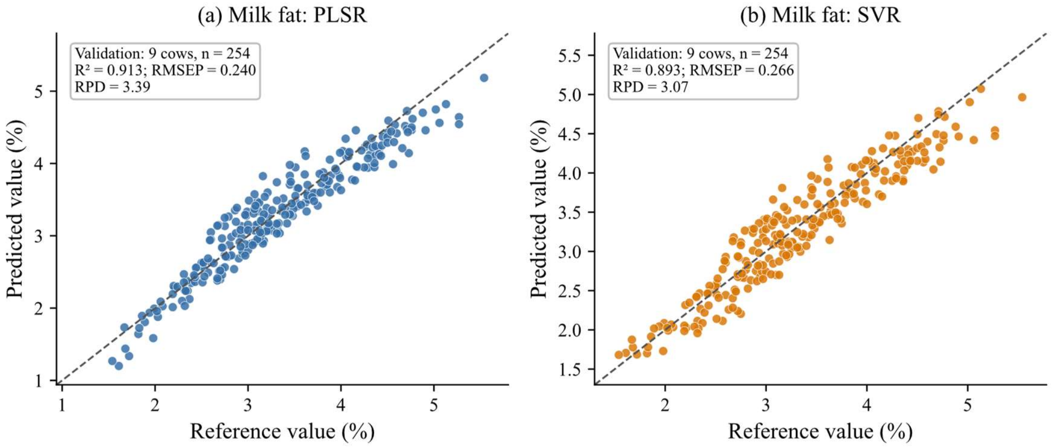

Figure S4. Validation of milk-fat prediction using (a) PLSR and (b) SVR.

Corn: paired cross-instrument moisture prediction

The corn dataset contained the same 80 samples measured on three instruments (m5, mp5 and mp6). A fixed 60/20 split of paired sample identities was used. Model selection and calibration used m5 spectra from the 60 calibration samples; the unchanged models were then applied to the same 20 validation samples measured with m5, mp5 and mp6. No transfer correction was used.

Dataset download address: https://eigenvector.com/wp-content/uploads/2019/06/corn.mat_.zip

Table S4. Moisture-prediction performance for paired corn samples measured on three instruments.

Model	Test instrument	Selected workflow	Performance
PLSR	m5 / mp5 / mp6	PLSR/Raw/LV=12	m5: R^2 0.997; RMSEP 0.021; RPD 19.01<br>mp5: R^2 −10.485; RMSEP 1.313; RPD 0.30<br>mp6: R^2 −11.342; RMSEP 1.361; RPD 0.29
SVR	m5 / mp5 / mp6	SVR/Raw/C=100<br>RBF; γ = 'scale'<br>ε = 0.05	m5: R^2 0.931; RMSEP 0.102; RPD 3.90<br>mp5: R^2 −1.644; RMSEP 0.630; RPD 0.63<br>mp6: R^2 −1.038; RMSEP 0.553; RPD 0.72

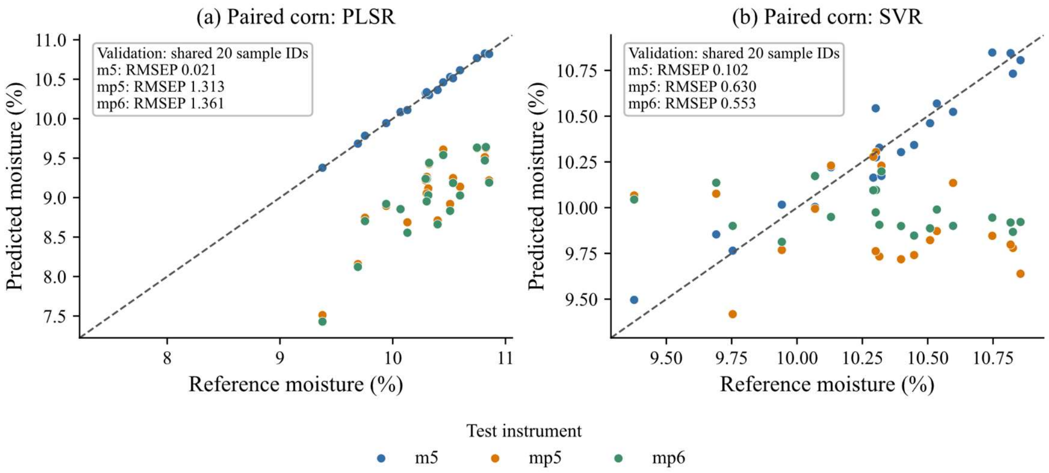

Figure S5. Validation results for corn moisture using (a) PLSR and (b) SVR..

Table S5. Representative natural-language task specifications.

Task	Natural-language specification
Tea data inspection	Inspect the four tea datasets. Verify instrument identity, sample state, spectral coordinate unit and direction, signal representation, class label, moisture target and calibration/validation partitions before modelling.
Raw spectra	Plot raw absorbance spectra for the four instrument–sample-state datasets using verified wavelength axes and English labels.
Calibration PCA	Perform PCA separately for each calibration dataset using unprocessed spectra. Produce score plots and PC1–PC5 explained-variance plots without using validation spectra.
Spectral preprocessing and models	Evaluate the predefined spectral preprocessing separately for each dataset and model family. Select preprocessing methods and model parameters using stratified four-fold cross-validation for classification and leave-one-product-out cross-validation for moisture prediction, restricted to the calibration set.
Classification	Compare PLS-DA and SVC and report accuracy, macro precision, macro recall and macro F1.
Moisture prediction	Compare PLSR and SVR and report R^2, RMSECV/RMSEP, MAE, bias, SEP and RPD.
Validation	Apply the selected models to the corresponding validation set. Report classification metrics and confusion matrices, and reference-versus-predicted and residual plots for moisture prediction.
Spectral variability	Using raw calibration spectra, calculate category-level STD RMS values and compare ground and unground samples separately for each instrument.
